# Permeability Evaluation Through Chitosan Membranes Using Taguchi Design

**DOI:** 10.3797/scipharm.1009-08

**Published:** 2010-10-21

**Authors:** Vipin Sharma, Rakesh Kumar Marwaha, Harish Dureja

**Affiliations:** Faculty of Pharmaceutical Sciences, M. D. University, Rohtak – 124 001, India

**Keywords:** Chitosan, Polymeric membrane, Permeation, Diclofenac diethylamine, Taguchi design

## Abstract

In the present study, chitosan membranes capable of imitating permeation characteristics of diclofenac diethylamine across animal skin were prepared using cast drying method. The effect of concentration of chitosan, concentration of cross-linking agent (NaTPP), crosslinking time was studied using Taguchi design. Taguchi design ranked concentration of chitosan as the most important factor influencing the permeation parameters of diclofenac diethylamine. The flux of the diclofenac diethylamine solution through optimized chitosan membrane (T9) was found to be comparable to that obtained across rat skin. The mathematical model developed using multilinear regression analysis can be used to formulate chitosan membranes that can mimic the desired permeation characteristics. The developed chitosan membranes can be utilized as a substitute to animal skin for *in vitro* permeation studies.

## Introduction

Transdermal drug delivery systems (TDDS) deliver therapeutic quantities of drug through the skin into the systemic circulation for their general effects. Several transdermal drug delivery systems have been developed and introduced into the market place since introduction of scopolamine in 1981 [1]. Evaluation of TDDS can be performed either using *in vivo* methods or by *in vitro* studies. Animal models (hairless rat, rabbit, guinea pig etc.) and human volunteers are generally used for *in vivo* studies [2]. It is almost often undesirable and non convenient to use human volunteers for routine quality control permeability tests due to ethical reasons. Animal models are however very useful in toxicology and mechanistic studies but extrapolation of the results to human *in vivo* situation should be done with utmost caution [3]. *In vitro* studies can be performed with human skin, animal skin and artificial membranes. Human skin *in vitro* models are most reliable but may be difficult to conduct due to scarcity of human skin and the effect of anatomical site, gender, age and race on the skin permeability [4]. The permeability characteristics between human skin and animal skin may differ due to the difference in skin permeation pathways, lipid content and variation in water uptake [5]. The difficulty in obtaining human/animal skin and variation in their permeability has led research workers to opt for artificial membranes. Moreover, the ethical concern against the use of animal/human skin makes polymeric membranes as an alternative model for evaluation of transdermal permeation. Polymeric membranes of chitosan have been developed by various researchers to imitate the permeability characteristics with the animal skin [6–8]. Artificial membranes have certain advantages like controlled composition, ease of preparation, availability of raw material, reproducible results over biological membranes.

Chitosan is a natural linear polysaccharide which is prepared from chitin by action of the enzyme chitin deacetylase. Chitosan is being widely used because of its biodegradability, biocompatibility and good film forming properties. Chitosan can be complexed with crosslinking agents like sodium citrate, sodium tripolyphosphate etc., which make chitosan a useful polymer for artificial membranes, microspheres, beads, wound dressings and nanoparticles. Diclofenac diethylamine, a topical non-steroidal anti-inflammatory drug, was used as a model drug for permeability evaluation through chitosan membranes.

In the present study, chitosan membranes capable of simulating permeation characteristics of diclofenac diethylamine across rat skin were prepared using Taguchi design. The objective behind the study was to identify the critical variables influencing the permeation characteristics through chitosan membranes.

## Materials and Methods

Chitosan, 95% deacetylated (Sigma Aldrich, Switzerland), Diclofenac diethylamine (Arion Pharmaceuticals, Chandigarh), Sodium tripolyphosphate (Thomas and Baker, Mumbai), Glacial Acetic Acid (Ranbaxy Fine Chemicals Limited, New Delhi), HPLC grade Methanol (Ranbaxy Fine Chemicals Limited, New Delhi) were used as received. All other reagents were of analytical grade.

### Taguchi Design

To study the effect of the process and formulation variables on permeation parameters of diclofenac diethylamine, the Taguchi design was applied. The Taguchi design utilizes orthogonal arrays from design of experiments theory to study a large number of variables with a small number of experiments. The use of orthogonal arrays significantly reduces the number of experimental configurations to be studied. Furthermore, the conclusions drawn from small scale experiments are valid over the entire experimental region spanned by the control factors and their settings [9–10]. To study three variables at three levels, Taguchi design revealed “L9” design, means that nine experiments are to be carried out. The study of three critical variables i.e. concentration of chitosan (X1), concentration of crosslinking agent, NaTPP (X2) and crosslinking time (X3) at 3 levels (low, medium and high) lead to formulation of nine chitosan membranes.

### Preparation of chitosan membranes

All the chitosan membranes were prepared using cast drying method as reported by Dureja *et al.*, [6]. Chitosan (powder) was dissolved in glacial acetic acid solution (2% v/v) to prepare chitosan solution (3–5 % w/v) and homogenized (2000 rpm). Then the solution was filtered through muslin cloth to remove debris and a portion was degassed by sonication at 25 °C for 20 min. The chitosan solution was cast on a polycarbonate petri dish and dried at 45 °C for 48 h. The dried membranes were crosslinked by dipping in a sodium tripolyphosphate solution (10 ml of 5–10% w/v) for 15–45 min. The membranes were further washed with distilled water to remove excess NaTPP. Freshly crosslinked membranes that were insoluble in phosphate buffer pH 7.4 (receptor solution) for more than 48 hrs were used for *in vitro* permeation studies. The formulation of chitosan membranes using Taguchi design (T1–T9) are shown in Table 1.

### Preparation and conditioning of rat skin

Rat skin was obtained after sacrificing the animal by inhalation of excess chloroform. The protocol was approved by Institutional Ethical Committee. Dorsal skin portion of albino Wistar rats was shaved with an electrical hair clipper and excised after sacrificing. To remove small hairs, depilatory cream was applied over the skin of rat for half an hour and then washed with distilled water. Freshly prepared rat skin was washed with phosphate buffer solution pH 7.4 and conditioned under stirring in a receptor solution for 4 h before initiating *in vitro* permeation experiments.

### In vitro Permeation Study

Franz diffusion cell apparatus (Hanson’s Research, USA) was used for *in vitro* permeation studies. Chitosan membranes or rat skin were clamped between donor and receptor compartments. Donor compartment was loaded with saturated solution of diclofenac diethylamine. The receptor compartment contained phosphate buffer pH 7.4 with formalin solution [0.1%w/v of 37–41% v/v] to prevent microbial growth. Stirring of receptor fluid was performed at 600 rpm and at 37±05 °C. Aliquots (0.5 ml) were withdrawn at various intervals and immediately analyzed for diclofenac diethylamine by HPLC (Agilent Technologies 1200 series, Germany) using eclipse XDB-C_18_ column (4.6×150 mm), and UV detector (Dual wavelength) at 221nm by modifying the method as reported by Mulgund *et al.,* [11]. A mixture of methanol and water (70:30) at flow rate of 1.0 ml min^−1^ was used as mobile phase for diclofenac diethylamine.

## Results and Discussion

Taguchi design was used to study the effect of concentration of chitosan, concentration of NaTPP and CL time on permeation parameters of diclofenac diethylamine (DDEA). Nine chitosan membranes (batch T1–T9) were developed (Table 1) and cumulative permeation of DDEA solution through these chitosan membranes was calculated. The permeation parameters i.e. flux and lag time was calculated from the linear portion of the graph (cumulative amount of drug permeated versus time). The signal to noise ratio (SN number) was calculated for each experiment to determine the effect of each variable on the flux. The calculated flux, lag time, thickness and SN value of chitosan membranes (batches T1–T9) are presented in Table 1. Ranking of factors (Table 2) was determined using average SN value of each factor.

Taguchi design ranked the concentration of chitosan as the most important factor influencing the permeation parameters of DDEA through chitosan membranes followed by concentration of NaTPP (crosslinking agent). The flux value increases at low levels of chitosan concentration, concentration of NaTPP and CL time. However, the flux was found to decrease with high values of chitosan concentration, i.e. the value of flux decreases as the concentration of chitosan increases. At high values of CL time, as the concentration of chitosan increases, the value of flux decreases. At low values of CL time, the lag time values were found to be low. The lag time was found to be lowest in case of low value of CL time with mid value of concentration of chitosan, whereas lag time was highest in case of mid values of CL time and with high value of concentration of chitosan. The high level of concentration of chitosan causes high values of lag time except at low values of CL time. Similarly, high value of CL time with high concentration of chitosan causes higher lag time except at low concentration of chitosan.

**Fig. 1.**
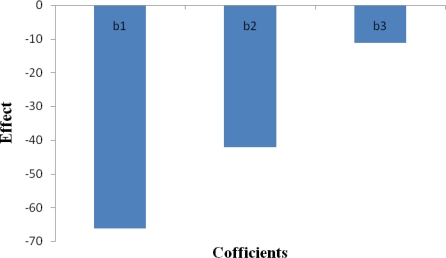
Effect plot of coefficients of flux

To determine the magnitude of contribution of different factors towards flux and lag time, multiple linear regression analysis was also performed. The real values of the factors were transformed to facilitate orthogonality of results and easy calculations. The effect plot of coefficients of flux is shown in Figure 1.

The mathematical model developed from multiple linear regression analysis to estimate flux can be represented as:

Y=120.9−66.3 X1−42.183 X2−11.133 X3

where Y= flux (μg.cm^−2^.sec^−1^), X1= Concentration of chitosan (%w/v), X2 = Concentration of NaTPP (%w/v), X3 = CL time (sec).

ANOVA was applied on the flux (Table 3) to study the fitting and significance of the model. F-test was carried out to compare the regression mean square with residual mean square, the ratio F= 6.170 show the regression to be significant. The estimated model, therefore, may be used to estimate the flux. The developed model can be further utilized to develop chitosan membranes of desired flux.

The *in vitro* evaluation of newly developed transdermal drug delivery system is primarily performed by the researchers using rodents/rats due to their availability and ease of estimation. Therefore, the permeation parameters of diclofenac diethylamine from chitosan membranes were compared with dorsal skin of Wistar rats. It was observed that only batch T9 has comparable permeation characteristic of diclofenac diethylamine solution as through rat skin with a correlation coefficient of R= 0.86. The low value of correlation coefficient may be attributed to less number of measured data points. The mathematical model developed using multilinear regression can be further utilized to develop a chitosan membrane which will exactly mimic the permeation characteristics with rat skin.

## Conclusion

Transdermal drug delivery system has become established as an effective alternative during the past decades. The ethical concern against the use of animals or human volunteers makes polymeric membranes as an alternative model for evaluation of transdermal permeation. Chitosan membranes were developed using Taguchi design to mimic the permeability of diclofenac diethylamine through rat skin. Taguchi design ranked the concentration of chitosan as the most important factor affecting the permeation of DDEA through crosslinked chitosan membranes. The optimized chitosan membrane (batch T9) was found to have comparable permeation characteristics. The mathematical model developed can be used to formulate chitosan membranes with simulated permeation characteristics. The critical variables can be further optimized experimentally to simulate the permeation characteristics of DDEA through other animal skins or human skin. These membranes also offer significant advantages in terms of their ready availability, uniformity, tensile strength and chemical purity therefore; these membranes should be utilized for *in vitro* permeation study of TDDS.

## Authors’ Statements

### Competing Interests

The authors declare no conflict of interest.

### Animal Rights

The institutional and (inter)national guide for the care and use of laboratory animals was followed. See the material and methods part for details.

## Figures and Tables

**Tab. 1.** Formulation of chitosan membrane (T1–T9) and permeation parameters

**Batch No.**	**X1 (%w/v)**	**X2 (%w/v)**	**X3 (min)**	**Flux±SD (μg.cm^−2^.sec^−1^)**	**Lag Time × 60±SD (sec)**	**Thickness ± SD (cm)**	**SN Value**
T1	1(3)	1(5)	1(15)	283.0±1.02	6.15±0.05	0.10±0.001	35.209
T2	1(3)	2(7.5)	2(30)	115.8±0.70	19.74±0.03	0.10±0.004	24.980
T3	1(3)	3(10)	3(45)	162.5±0.82	6.74±0.02	0.10±0.005	29.406
T4	2(4)	1(5)	2(30)	182.7±1.05	7.62±0.05	0.08±0.002	34.566
T5	2(4)	2(7.5)	3(45)	111.0±0.85	42.42±0.08	0.09±0.006	29.663
T6	2(4)	3(10)	1(15)	69.6±0.94	4.69±0.06	1.05±0.008	24.658
T7	3(5)	1(5)	3(45)	65.1±0.82	44.49±0.03	0.10±0.001	25.764
T8	3(5)	2(7.5)	1(15)	52.8±1.13	16.82±0.04	0.09±0.004	21.322
T9	3(5)	3(10)	2(30)	45.6±1.02	47.58±0.06	0.07±0.007	20.239
Rat skin	–	–	–	36.6±0.91	52.68±0.09	0.08±0.07	–

X1= concentration of chitosan, X2= concentration of NaTPP, X3= crosslinking time. Values in the bracket indicate real values.

**Tab. 2.** Ranking of factors

**Level**	**X1**	**X2**	**X3**
1	29.865	31.846	27.663
2	29.629	25.321	26.595
3	22.441	24.767	28.277
Δ	7.424	7.097	1.682

**Rank**	**1**	**2**	**3**

**Tab. 3.** ANOVA of the regression (flux)

	**Degree of freedom**	**Sum of Squares**	**Mean Square**	**F**	**F-significance**
Regression	3	37794.45	12598.15	6.170233	0.03911
Residual	5	10208.81	2041.762		
Total	8	48003.26			
